# Asymptomatic Carriage and Antimicrobial Resistance of *Salmonella* in Humans and Poultry in Rural Burkina Faso: Phenotypic and Genotypic Profiles and Associated Risk Factors

**DOI:** 10.3390/microorganisms14020294

**Published:** 2026-01-27

**Authors:** Ibrahima Karama, Daniel Valia, Sandeep Tamber, Christian Marc Tahita, Palpouguini Lompo, Sibidou Yougbare, Mary Rao, Annika Flint, Kelly Weedmark, Zakaria Garba, William Alix L. Tiendrebeogo, Albert Patrick Vokouma, Eric Wendpouiré Tiendrebeogo, Georges Somé, Marjan Peeters, Jan Jacobs, Marianne A. B. van der Sande, Henri Gautier Ouédraogo, Halidou Tinto, Nicolas Barro

**Affiliations:** 1 LabESTA, École Doctorale Sciences et Technologies, Université Joseph KI-ZERBO, Ouagadougou 03 BP 7021, Burkina Faso; karamaibrahima@gmail.com; 2 Unité de Recherche Clinique de Nanoro (URCN), Institut de Recherche en Sciences de la Santé (IRSS), Centre National de la Recherche Scientifique et Technologique (CNRST), Ouagadougou 11 BP 218, Burkina Faso; 3 Bureau of Microbial Hazards, Food and Nutrition Directorate, Health Canada, Ottawa, ON K1A 0K9, Canadakelly.weedmark@hc-sc.gc.ca (K.W.); 4 Department of Clinical Sciences, Institute of Tropical Medicine, 2000 Antwerp, Belgium; 5 Department of Microbiology, Immunology and Transplantation, KU Leuven, 3000 Leuven, Belgium; 6 Department Global Public Health & Bioethics, Julius Center, 3584 CX Utrecht, The Netherlands

**Keywords:** *Salmonella enterica*, asymptomatic carriage, antimicrobial resistance, poultry and human infections, resistance and virulence genes, risk factors, Burkina Faso

## Abstract

Food-borne diseases affect nearly 10% of the global population annually, with *Salmonella* being a major cause, particularly impacting children, the elderly, and populations in low- and middle-income countries. This study aimed to assess the prevalence, serotype distribution, antibiotic resistance profiles, and genetic determinants of resistance and virulence of *Salmonella enterica* in humans and poultry in the Nanoro health district. A community-based cross-sectional study involving humans and poultry was conducted in the Nanoro health district. Fresh stool samples (human and poultry cloacal/cecal) were collected, transported under sterile conditions, and processed within two hours using standard bacteriological methods. Phenotypic antibiotic resistance was determined by the Kirby–Bauer disk diffusion method, and whole-genome sequencing (Illumina) identified serotypes, resistance genes, and virulence factors. Logistic regression analyzed associations between *Salmonella* carriage and host or environmental factors. *Salmonella enterica* carriage was detected in 8.7% of humans and 7.2% of poultry. Human isolates showed 24% resistance to cephalosporins, while poultry isolates showed 36.8% resistance. Resistance genes, including fosA7, qnrB19, and a cryptic aminoglycoside resistance gene, and virulence genes encoding T3SS-1 and T3SS-2, were detected in both hosts. Logistic regression indicated that residence in Sitaon and Zimidin was associated with ~70% lower odds of carriage (aOR = 0.3), while individuals aged 11–20 and 51–60 years had 2.8-fold higher odds. Carriage was also 60% higher during the rainy season. These findings suggest possible cross-transmission of *Salmonella* between humans and poultry and the circulation of resistant, potentially virulent strains in the community. Seasonal and age-related variations highlight environmental and behavioral influences on asymptomatic carriage. Integrated One Health surveillance and targeted hygiene interventions are essential to reduce *Salmonella* transmission and antimicrobial resistance in rural settings.

## 1. Introduction

*Salmonella*, a pathogenic bacterial genus, is responsible for a wide range of illnesses, including typhoid (enteric) fever and gastroenteritis. With more than 2500 known serovars, Salmonella represents a significant global public health concern, being widely distributed across human, animal, and environmental reservoirs, and increasingly associated with antimicrobial resistance at the human–animal–environment interface [1,2,3]. It has a high disease burden and can be associated with antimicrobial resistance (AMR), increasing its threat to human health [4]. Typhoidal *Salmonella* (TS) includes *Salmonella enterica* serovars *Typhi* and *Paratyphi* A, B, and C and is clinically distinct from non-typhoidal *Salmonella* (NTS). Non-typhoidal *Salmonella*, including serovars such as *S.* Enteritidis, *S.* Typhimurium and *S.* Gallinarum, is a major cause of foodborne gastroenteritis worldwide [5,6]. The main pathogenic *Salmonella enterica* strains in poultry are *S.* Gallinarum, which causes avian typhoid and high mortality in birds of all ages, and *S. pullorum*, which causes pullorum disease with very high mortality in young chicks during the first weeks of life [7]. NTS transmission to humans usually follows a ‘farm-to-fork’ pathway; some *Salmonella* serovars can survive in chilled meat, emphasizing proper cold chain management [1,8].

In sub-Saharan Africa, *Salmonella enterica* serovars Enteritidis and Typhimurium account for approximately 91% of reported African salmonellosis outbreaks [9]. The population at risk of severe outcomes or infection includes babies, young adults, and immunocompromised individuals, particularly those living with human immunodeficiency virus and/or malaria [10]. It was estimated that approximately 535,000 cases of invasive non-typhoidal salmonellosis and 77,500 deaths occurred in 2017 in the world. Of these, 422,000 cases (78.9%) and 66,500 deaths (85.9%) occurred in countries in sub-Saharan Africa [11]. The invasive form of NTS (iNTS) continues to pose a major challenge, with the 2021 Global Burden of Disease Study estimating over 500,000 global iNTS cases, the majority occurring in West Africa [12].

In both human and animal populations, chronic and asymptomatic carriers play a key role in maintaining transmission cycles and therefore complicating disease eradication efforts [13]. In poultry, *Salmonella enterica* often persists asymptomatically, enabling prolonged shedding into the environment and enhancing transmission among flocks, including via biofilm formation on farm surfaces [14,15]. Similarly, asymptomatic human carriers contribute to household or community spread [16,17].

The rising challenge of antimicrobial resistance (AMR) adds to the complexity of *Salmonella* management. Indeed effective antibiotics are now compromised by resistant strains, threatening treatment efficacy in both human and veterinary medicine [18,19,20,21].

Despite the public health importance of *Salmonella*, epidemiological and resistance data remain scarce in rural resource-limited settings such as Burkina Faso, where close human–animal interaction is common, and food production is frequently informal. *Salmonella*’s ability to colonize diverse hosts, including wildlife such as free-living turtles, further highlights its adaptability and public health challenge [22,23]. While some studies have explored avian *Salmonella* carriage and resistance in urban settings [22,24,25], data on rural populations remain limited. In several countries, including the United States, studies employing whole-genome sequencing and source-attribution modeling estimate that chicken alone may account for approximately one-third of sporadic human *Salmonella* cases, and up to ~70–75% when combining chicken with other high-confidence sources such as eggs and pork products [26]. Within the European Union, the contribution of laying hens and poultry meat/eggs to cases varies significantly between countries (e.g., approximately 42% of cases are attributed to laying hens in a multi-country European Union model) [26]. However, to date, there appears to be an absence of comparable quantitative attribution data from sub-Saharan Africa or Burkina Faso.

Prior investigations in Nanoro, a rural district of Burkina Faso, have revealed diverse *Salmonella* serotypes and high antibiotic resistance rates in strains from humans, animals, food, and the environment. However, the mechanisms underlying this antibiotic resistance and virulence in *Salmonella enterica*, including associated genetic determinants, remain poorly explored in this specific context [27]. In such settings, the management of *Salmonella* infection requires robust data to support surveillance, prevention, and control strategies. Therefore, this study aimed to assess the prevalence of asymptomatic *Salmonella* carriers among healthy humans and poultry in Nanoro, identify the *Salmonella* serotypes present, examine their resistance to antibiotics, and detect the related genetic markers of resistance and virulence.

## 2. Material and Methods

### 2.1. Study Site

This study was conducted in the Nanoro health district, central-west Burkina Faso, about 90 km from Ouagadougou. The district has 30 primary healthcare centers (PHCs), each covering a health catchment area. Stool samples were collected in two catchment areas: Nanoro (five villages: Nanoro, Gouroumbila, Poessi, Baziri, and Goulouré) and Nazoanga (three villages: Nazoanga, Zimidin, and Sitaon) [28], both embedded in the Clinical Research Unit of Nanoro (CRUN) Health and Demographic Surveillance System (HDSS) [29].

### 2.2. Study Design, Population, and Criteria for Selecting Participants; Sampling

This was a non-interventional, cross-sectional study conducted from May 2021 to May 2022 and was an ancillary embedded within the CABU pilot study (Community-level AntiBiotic use in Nanoro district Burkina Faso) [30]. Two study populations were defined: a human population and a poultry population.

The human population consisted of 1500 individuals for whom sociodemographic characteristics were collected using a standardized questionnaire (Appendix A and Appendix B). Participants were required to be healthy at the time of the survey; if ill, the visit was postponed until recovery, and all medical consultations in the preceding three months were recorded. Fresh stool samples were collected from consenting participants and stored in sterile containers.

The poultry population included birds owned by households participating in the human survey as well as poultry obtained from local vendors. A total of 528 chicken samples were collected. Household-owned poultry (500 households) were sampled using cloacal swabs collected in Amies transport medium (Deltalab. Plz. Verneda 1, Pol Ind La Llana 08191 Rubí, Barcelona, Spain). Poultry vendors were not considered a separate human study population but were included solely as focal points for poultry sampling, as they aggregate birds originating from multiple households and represent a major source of poultry supply within the community. For vendor-associated poultry, birds were sacrificed at the point of sale and cecal contents were collected in preservative-free sterile containers.

All samples were kept at 2–8 °C and transported to the laboratory within two hours of collection.

### 2.3. Isolation, Identification and Antibiotic Susceptibility Testing

The stool samples were analyzed in the microbiology laboratory of the Clinical Research Unit of Nanoro. Approximately 10 g of stools was inoculated into in 5 mL of selenite enrichment broth (Liofilchem^®^ s.r.l Via Scozia, 64026 Roseto degli Abruzzi (TE), Italy). After incubation for 18–24 h at 35 ± 2 °C, 10 µL of the enrichment broth was plated on CHROMagar^TM^ *Salmonella* (CHROMagar 29 Av. George Sand, 93210 Saint-Denis, France) for the detection of suspected *Salmonella* colonies. The plated agar was incubated 18–24 h at 35 ± 2 °C. Poultry cecum and rectal samples were initially pre-enriched in 5 mL of peptone water and then incubated for 18–24 h at 35 ± 2 °C. Subsequently, 1 mL was transferred into 5 mL of selenite broth for targeted enrichment at 41 °C for 48 h. Following this, 10 µL of the enriched broth was plated onto CHROMagar™ *Salmonella* and then incubated for 18–24 h at 35 ± 2 °C. Presumptive *Salmonella*, identified by purple colonies, was then isolated and identified using the API 20E system. Our strains were stored in STGG (skim milk–Tryptone–Glucose–Glycerol) broth at −80 °C for further analysis and as copies. Human stool samples were directly enriched in selenite broth, as stools generally contain a higher background level of enteric bacteria and Salmonella, and selenite enrichment is well established for selective recovery from human fecal samples. In contrast, poultry cecal and cloacal samples were first pre-enriched in non-selective buffered peptone water to allow recovery of stressed or low-abundance Salmonella cells potentially affected by environmental conditions or slaughter procedures. This was followed by selective enrichment in selenite broth, in accordance with standard bacteriological protocols for animal samples.

Antibiotic susceptibility testing (AST) was performed using the disc diffusion method according to the Clinical and Laboratory Standard Institute (CLSI) to assess bacterial resistance and the results were interpreted using the CLSI guidelines, version 2023 [31].

### 2.4. DNA Extraction and Illumina Sequencing

Cells were retrieved from storage at −80 °C and plated onto tryptic soya agar (TSA). Plates were incubated at 35 °C overnight and 1 µL loopful of cells was collected in 0.4 mL of 1x Zymo DNA/RNA Shield (Cedarlane, Burlington, VT, Canada) and held at 4 °C. DNA extractions were performed using the Zymo Quick-DNA HMW MagBead kit (Cedarlane) according to manufacturer protocols (RNAseA treatment without enzymatic lysis). Illumina libraries were constructed using the NexteraXT DNA Library Preparation Kit and Nextera DNA UD Indexes according to the manufacturer’s instructions (Illumina Inc., San Diego, CA, USA). Paired-end Illumina sequencing was performed on a MiSeq instrument (v3 chemistry, 2 × 300 bp) according to manufacturer instructions (Illumina Inc.).

### 2.5. Bioinformatic Analysis

#### 2.5.1. Read Processing, De Novo Genome Assembly, and Taxonomic Assignment

Illumina reads were processed using BBMap v38.26 (sourceforge.net/projects/bbmap) to remove adapters and filter low-quality reads (BBDuk), and correct errors (Tadpole). De novo assemblies were generated using SKESA v2.4.0 [32] and error corrected using Pilon v1.23 [33]. Assemblies were analyzed with QUAST v5.0.2 (github.com/ablab/quast) and taxonomically assigned using Mash v2.2.1 [34] and the highest RefSeq (v93, ncbi.nlm.nih.gov/refseq) identities of >0.8 (excluding hits containing the words phage or plasmid). Analysis tools were used with default settings unless noted.

#### 2.5.2. In Silico Typing, Antimicrobial Resistance, and Virulence Factor Identification

Multi-locus sequence typing was performed using MLST (v2.23.0, github.com/tseemann/mlst) and Enterobase database (v2023-11-20) [35,36] using the --scheme senterica_achtman_2 and default settings. SISTR (v1.1.3) [37] was used for serovar prediction using the --qc option and default settings. Antimicrobial resistance genes were identified using the assembled genomes and ResFinder (v4.3.1) [38]. ResFinder was run using the -acquired option, minimum coverage length, and percent identity settings of 60 and 90 percent, respectively (-l 0.6 and -t 0.9), and the ResFinder database (v2023-05-25). Pathogenicity islands were identified using SPIFinder (v2.0) [39] and SPIFinder database (v2020-12-04, bitbucket.org/genomicepidemiology/spifinder_db) using minimum coverage length and threshold settings of 60 and 90 percent, respectively (-l 0.6 and -t 0.9). Virulence genes were identified using ABRicate (v1.0.1, github.com/tseemann/abricate) and the Virulence Factor Database (v2023-11-04) [40] using default settings. All parameters have been summarized in Supplementary Table S1.

### 2.6. Statistical Analysis

Statistical analyses were performed using STATA software version 17, 2021. Quality control checks were performed prior to the statistical analysis. Outliers from measurement or data entry errors were corrected, and those likely to affect the analyses were excluded. Categorical variables were summarized using counts and percentages, whereas continuous variables were summarized using medians and interquartile ranges (IQR), when appropriate. Comparisons between the outcome variable and continuous variables were performed using the Wilcoxon–Mann–Whitney test, while Pearson’s chi-square or Fisher’s exact tests were applied for categorical variables, as appropriate. A binary logistic regression analysis was conducted to identify determinants of salmonellosis. Odds ratios (ORs) and their 95% confidence intervals (95% CI) were reported. Multivariable models were built using a stepwise elimination procedure, including variables with a nominal *p*-value < 0.20 in the univariate analysis. A *p*-value < 0.05 was considered statistically significant. Missing values were handled using mode imputation after comparing several approaches, including the creation of a missing category and multiple imputations. This method was applied for the multivariate analysis to preserve sample size and maintain consistency of the estimates.

### 2.7. Ethics

The study protocol was approved by the national ethics committee of Burkina Faso with approval number 2021-03-052 at deliberations on 10 March 2021 and the Institute of Tropical Medicine Institutional Review Board ref. 1397/20. Written informed consent was obtained from all study participants aged 18 years and above. For participants aged 14 to <18 years, oral assent was obtained in addition to written informed consent from their parents or caregivers. For participants younger than 14 years, written informed consent was obtained directly from their parents or caregivers.

## 3. Results

### 3.1. Sociodemographic Characteristics by Salmonella enterica Carriage in Humans

A total of 1500 individuals or households were enrolled in the study, of which 1393 were included in the final analysis of the human population; the remaining participants were excluded due to refusal of consent or missing data. Among the poultry samples, 500 out of 528 were collected from households participating in the human survey, while the remaining 28 originated from poultry vendors. Due to small numbers of events in certain categories, the findings should be interpreted with caution. The study population was predominantly young, with a median age of 13 years with 39 (IQR; 5.0–44.0). Asymptomatic *Salmonella* carriers tended to be slightly older, with a median age of 15 years. Although the median age among asymptomatic carriers was higher, this difference was not statistically significant (*p* = 0.480). In contrast, the distribution of *Salmonella* carriage across age groups showed a statistically significant variation. Geographical distribution varied considerably across study sites, with participants from Nanoro representing the largest proportion of the study population. Detailed demographic characteristics are summarized in Table 1.

### 3.2. Prevalence of Asymptomatic Carriage and Distribution of Salmonella Serotypes

Out of the 1393 people enrolled in this study, 121 were identified as *Salmonella* carriers, resulting in an asymptomatic carriage prevalence of 8.7% (121/1393). Among participants owning poultry, fecal samples revealed that 7.2% (38/528) of the poultry was *Salmonella* positive. The distribution of *Salmonella* prevalence differed significantly between villages (*p* < 0.001). Nanoro Center recorded the highest proportion of *Salmonella*-positive individuals, accounting for 42.5% (51/121) of all asymptomatic carriers, whereas Poessi showed the highest *Salmonella* carriage rate among poultry (15.8%, 6/38). In contrast, the villages of Zimidin and Sitaon had the lowest prevalence rates, 4.2% (5/121) and 2.5% (3/121), respectively (*p* = 0.036 and *p* = 0.050). Most *Salmonella* isolates were detected between July and December, corresponding to the transition between the rainy and dry seasons. Table 2 shows the prevalence and distribution of *Salmonella* isolates from asymptomatic carriers and their poultry in the Nanoro health district.

### 3.3. Frequent Sequence Type (ST)

Most isolates, whether from asymptomatic humans or poultry, remain untyped (-). Among the typed isolates, ST516 is the only sequence type found in both populations, while the other types are specific to each source (ST513 and ST1737 in humans, ST3031 in poultry). However, each type represents only a small fraction of the total, highlighting high genetic diversity and the absence of a clearly dominant type.

### 3.4. Antibiotics Resistances and Virulence Genes

Several antibiotics were used for susceptibility testing against *Salmonella* strains. There was a high prevalence of cephalosporin resistance in humans and poultry (24.0% and 36.8% respectively). Multidrug-resistant strains (isolates that show non-susceptibility acquired to at least one agent in three or more different classes of antimicrobial drug) were detected only in humans, at 1.7%. Details are given in Table 3. Poultry is a reservoir of resistance to antibiotics for human use, since resistant strains can be found there.

This study enabled us to highlight genotypic resistance to certain antibiotics commonly used to treat salmonellosis, as well as to those not used at all for these strains. For example, we identified a cryptic resistance gene to aminoglycosides, fosfomycin, and quinolones shown in Table 4. Human and poultry isolates carried fosfomycin resistance genes when this antibiotic is not used for salmonellosis treatment. We have also identified the presence of virulence genes in *Salmonella* isolates from both humans and poultry, notably aac(6’)-Iaa (cryptic aminoglycoside resistance gene) and SPI1 to 5 (Table 4).

### 3.5. PointFinder Results

PointFinder analysis revealed point mutations in genes associated with quinolone resistance. A well-characterized resistance-associated substitution in *gyrA* was detected and was consistent with phenotypic resistance to quinolones and fluoroquinolones.

In addition, the *parC T57S* substitution was frequently identified among the isolates; however, this mutation was not systematically associated with phenotypic resistance to quinolones. The genes found are summarized in Supplementary Table S2.

### 3.6. Associated Risks Factors of Salmonella Carriage

Overall, the findings indicate that *Salmonella* carriage is influenced by a combination of geographic, demographic, and seasonal factors. In the multivariate analysis of the human population, significant differences in asymptomatic *Salmonella* carriage were observed between villages. Specifically, individuals from Sitaon and Zimidin exhibited a markedly lower risk of carriage (adjusted odds ratio [aOR] 0.3; 95% CI [0.1–0.9]), with *p*-values of 0.050 and 0.036, respectively, compared with individuals from other villages. In contrast, participants from Nanoro showed a slightly higher risk (aOR 1.5 [0.7–3.5]), although this association did not reach statistical significance. Individuals aged 11–20 years and 51–60 years had an increased likelihood of *Salmonella* carriage (aOR 2.8 [1.0–8.9]). Children aged 0–10 years and adults aged 61–70 years also showed a higher risk of carriage (≈60%), but these associations were not statistically significant. Seasonal variation (*p*-value = 0.008) further influenced carriage risk. The rainy season was associated with a 60% increase in the likelihood of carriage among humans (aOR 1.6 [1.0–2.7]), highlighting the potential impact of environmental factors on transmission. Among the poultry population, the month of May was associated with a substantial increase in *Salmonella* carriage (aOR 5.3), suggesting temporal patterns in bacterial prevalence within animal reservoirs. A detailed summary of all significant determinants is presented in Table 5.

## 4. Discussion

This study provides comprehensive insights into *Salmonella* carriage in rural Burkina Faso in humans and poultry in their immediate environment, highlighting substantial geographic and demographic variability. The prevalence of *Salmonella* was 8.7% in humans and 7.2% in poultry, higher than reports from Ghana, Togo, and Nigeria (0.8–7.5%) [41] and comparable to some regions in Kenya (0.4–13.1%) [42] and Mexico (11.4% in children under five) [43]. These variations likely reflect differences in environmental conditions, socio-economic factors, urbanization, poultry production systems, hygiene practices, and methodological approaches [6,44,45,46]. Within our study, carriage was higher in the 6–64 age group, possibly due to increased occupational and food-related exposure and a more mature gut microbiota, whereas children under five also showed notable carriage relative to previous studies in Kenya [42].

In poultry, prevalence in our study was lower than in previous reports in Burkina Faso, which ranged from 9.9% to 63.5% in urban centers [47,48,49], and lower than in neighboring West African countries (29–47%) [50,51,52]. Differences with developed countries were also notable, with lower or comparable prevalence reported in Europe (8.6–22.7%) [53,54] and North America (6.5–26.9%) [55,56], whereas Asia showed highly variable rates (18–40%) depending on local food safety controls [57]. These findings suggest that *Salmonella* circulation is influenced by poultry production systems, biosecurity measures, density of animal and human populations, slaughtering practices, and local hygiene conditions. Seasonal variations, particularly during the rainy season, may further promote bacterial survival and environmental dissemination [58,59].

Phenotypic antimicrobial resistance (AMR) was higher in human isolates than in poultry, with resistance observed against cephalosporins, particularly ceftazidime, alongside evidence of multidrug resistance (MDR). Corresponding resistance genes, including bla_TEM, tetA, and sul1, were detected in both humans and poultry, although discrepancies between phenotype and genotype likely reflect gene expression variability or regulatory mechanisms [52,60,61,62,63,64]. Resistance to fosfomycin, not used clinically for salmonellosis, was also identified, highlighting the potential for emerging MDR and the need for rational antimicrobial use in livestock [65,66,67]. These findings have clinical and public health implications, as asymptomatic carriers can act as reservoirs for resistant strains, facilitating transmission through food and environmental pathways.

Genomic analyses revealed a core virulome, consisting of virulence genes broadly conserved across *Salmonella* isolates, including pathogenicity islands SPI-1 to SPI-5, SPI-9, SPI-13, SPI-14, and fimbriae/pili genes [68,69]. The presence of these genes in asymptomatic carriers underscores the role of host factors and gene regulation in determining infection outcomes, beyond the acquisition of novel virulence determinants. The co-occurrence of resistance and virulence genes further emphasizes the risk of genetic exchange and the potential for zoonotic transmission, reinforcing the need for integrated interventions [70,71]. Mutations in *gyrA* identified in this study are well-recognized determinants of quinolone and fluoroquinolone resistance in *Salmonella*, due to alterations in the quinolone resistance-determining region (QRDR) of DNA gyrase, with common substitutions at positions S83 and D87 reported worldwide [72,73]. In contrast, although frequently detected, the *parC* T57S substitution was not consistently associated with phenotypic resistance, supporting recent reports suggesting a limited or unclear role in quinolone resistance [74]. These findings highlight the importance of integrating genomic and phenotypic data for accurate interpretation of quinolone resistance mechanisms.

Demographic and environmental factors significantly influenced carriage in humans. Residents of Sitaon and Zimidin had a lower risk (aOR = 0.3; *p* < 0.05), while individuals aged 11–20 and 51–60 years exhibited higher risk. No significant association with sex was observed. These patterns suggest that local hygiene practices, environmental exposure, and age-related behaviors contribute to colonization. In poultry, although associations were not statistically significant, a trend toward increased carriage during the rainy season supports the hypothesis of environmental and zoonotic influences. Collectively, these findings highlight the importance of integrated One Health approaches that combine human, animal, and environmental surveillance to limit transmission within communities [6,46,75]

This study has several limitations. Its cross-sectional design prevents establishing causal relationships, and testing a single stool sample per participant may underestimate actual carriage. Finally, some confidence intervals were wide, reflecting low precision of the estimates due to small numbers of events in certain categories. Therefore, these results should be interpreted with caution. Despite these limitations, the study provides valuable baseline data on *Salmonella* prevalence, resistance, and virulence in both humans and poultry. Future research should employ longitudinal designs, larger and concomitant sample sizes, and extended genomic analyses to better understand the dynamics of resistance gene dissemination and zoonotic transmission. Strengthened surveillance, targeted interventions, improved poultry biosecurity, and judicious antimicrobial use remain essential to limit the spread of resistant and virulent *Salmonella* strains, particularly in high-risk rural areas.

## 5. Conclusions

This study demonstrates that asymptomatic carriage of *Salmonella* occurs in both humans and poultry in Burkina Faso, with a notable prevalence of antibiotic-resistant and multidrug-resistant strains. Resistance genes, particularly those conferring resistance to cephalosporin, fosfomycin, and others, were frequently detected. The presence of asymptomatic carriers represents a significant public health concern, as humans and animals can act as reservoirs for transmission, potentially leading to outbreaks and impacting food safety and local economies. These findings highlight the need for integrated surveillance across human, poultry, and environmental samples, alongside targeted hygiene education in high-prevalence communities and antibiotic stewardship in both human and veterinary sectors. Implementing such measures is crucial to mitigate the risk of *Salmonella* transmission, curb the spread of multidrug-resistant strains, and protect public health in Burkina Faso.

## Figures and Tables

**Table 1:** Sociodemographic and environmental characteristics distribution by *Salmonella enterica* carriage in humans.

Sociodemographic Characteristics	Total N_1_ = 1393	Negative 1272 (91.3%)	Positive 121 (8.7%)	*p*-Value
**Villages**				<0.001
Baziri	90 (6.5%)	81 (6.4%)	9 (7.5%)	
Goulouré	133 (9.6%)	123 (9.7%)	10 (8.3%)	
Gouroumbila	39 (2.8%)	36 (2.8%)	3 (2.5%)	
Nanoro	354 (25.4%)	303 (23.8%)	51 (42.5%)	
Nazoanga	391 (28.1%)	364 (28.6%)	27 (22.5%)	
Poessi	126 (9.1%)	114 (9.0%)	12 (10.0%)	
Sitaon	104 (7.5%)	101 (7.9%)	3 (2.5%)	
Zimidin	156 (11.1%)	150 (11.8%)	6 (5.02%)	
**Season**				0.011
Dry season	502 (36.0%)	456 (35.8%)	46 (38.0%)	
Rainy Season	198 (14.2%)	171 (13.4%)	27 (22.3%)	
Missing values	693 (49.7%)	645 (50.7%)	48 (39.7%)	
**Age in years (median and IQR)**	13.0 (39.0)	13.0 (38.0)	15.0 (40.5)	0.480 +
**Age group in years**				0.031 *
0–5	421 (30.2%)	390 (30.7%)	31 (25.8%)	
6–64	855 (61.4%)	770 (60.5%)	85 (70.8%)	
65–100	116 (8.3%)	112 (8.8%)	4 (3.3%)	
**Sex**				0.410
Female	746 (53.6%)	686 (53.9%)	60 (50.0%)	
Male	646 (46.4%)	586 (46.1%)	60 (50.0%)	

N_1_: Number of individuals in the human population included in the study. Results are given as medians (IQR) or n (column percent). + Wilcoxon-Mann-Witney test was performed for continuous variables when the conditions were met. Pearson chi-square’s test was performed for categorical variables. * Fisher’s Exact test was performed when the conditions were met.

**Table 2:** Isolated serotypes and serovar distribution in human and poultry.

Carriage of *Salmonella enterica*	Human N_1_ = 1393	Poultry N_2_ = 528
**Isolation of *Salmonella* strains**	**121 (8.7%)**	**38 (7.2%)**
*Abaetetuba*	3 (2.5%)	0 (0.0%)
*Adelaide*	1 (0.8%)	0 (0.0%)
*Agona*	3 (2.5%)	0 (0.0%)
*Amoutive*	1 (0.8%)	0 (0.0%)
*Bietri*	1 (0.8%)	0 (0.0%)
*Braenderup*	1 (0.8%)	0 (0.0%)
*Brancaster*	2 (1.7%)	0 (0.0%)
*Bredeney*	6 (5.0%)	1 (2.6%)
*Chandans*	1 (0.8%)	0 (0.0%)
*Chester*	1 (0.8%)	0 (0.0%)
*Cubana*	2 (1.7%)	0 (0.0%)
*Derby*	6 (5.0%)	10 (26.3%)
*Drac*	2 (1.7%)	1 (2.6%)
*Eastbourne*	3 (2.5%)	1 (2.6%)
*Edinburg*	1 (0.8%)	0 (0.0%)
*Enterica*	4 (3.3%)	1 (2.6%)
*Essen*	1 (0.8%)	1 (2.6%)
*Friedrichsfelde*	1 (0.8%)	0 (0.0%)
*Gaminara*	7 (5.8%)	0 (0.0%)
*Give*	7 (5.8%)	2 (5.3%)
*Gloucester*	1 (0.8%)	0 (0.0%)
*Hato*	1 (0.8%)	0 (0.0%)
*Ituri*	1 (0.8%)	0 (0.0%)
*Kaapstad*	1 (0.8%)	1 (2.6%)
*Kentucky*	1 (0.8%)	0 (0.0%)
*Kokomlemle*	1 (0.8%)	0 (0.0%)
*Korlebu*	4 (3.3%)	0 (0.0%)
*Miami*	0 (0.0%)	2 (5.3%)
*Monschaui*	2 (1.7%)	0 (0.0%)
*Montevideo*	0 (0.0%)	1 (2.6%)
*Muenster*	4 (3.3%)	1 (2.6%)
*Nima*	2 (1.7%)	0 (0.0%)
*Nottingham*	5 (4.1%)	0 (0.0%)
*Oranienburg*	1 (0.8%)	0 (0.0%)
*Poona*	10 (8.3%)	1 (2.6%)
*Rideau*	2 (1.7%)	0 (0.0%)
*Rissen*	1 (0.8%)	2 (5.3%)
*Saintpaul*	2 (1.7%)	0 (0.0%)
*Schwarzengrund*	2 (1.7%)	2 (1.7%)
*Senftenberg*	0 (0.0%)	1 (2.6%)
*Souza*	6 (5.0%)	0 (0.0%)
*Strathcona*	0 (0.0%)	1 (2.6%)
*Tennessee*	2 (1.7%)	0 (0.0%)
*Teshie*	2 (1.7%)	0 (0.0%)
*Tilene*	1 (0.8%)	0 (0.0%)
*Typhimurium*	6 (5.0%)	1 (2.6%)
*Urbana*	3 (2.5%)	0 (0.0%)
*Vilvoorde*	1 (0.8%)	0 (0.0%)
*Wagadugu*	0 (0.0%)	1 (2.6%)
*untyped*	5 (4.1%)	9 (23.7%)
**Isolation of *Salmonella* strains**		
**serovar clinics**		
Typhoid serovars	0 (0.0%)	0 (0.0%)
Non-typhoidal serovars	70 (57.9%)	27 (71.1%)
Rare Serovars	51 (42.1%)	11 (28.9%)
**Type of serovar**		
Serovars of human carriage	41 (33.9%)	14 (36.8%)
Serovars linked to animals	80 (66.1%)	24 (63.2%)

N_1_: Number of individuals in the human population included in the study. N_2_: Number of poultry included in the study.

**Table 3:** Antibiotic resistance profiles distribution in human and poultry.

Carriage of *Salmonella enterica*	Human N_1_ = 121	Poultry N_2_ = 38
Ampicillin (10 µg)	2 (1.7%)	0 (0.0%)
Amoxicillin and clavulanic acid (20/10 µg)	1 (0.8%)	0 (0.0%)
Piperacillin/tazobactam (100/10 µg)	0 (0.0%)	0 (0.0%)
Ceftriaxone (30 µg)	7 (5.8%)	1 (2.6%)
Ceftazidime (30 µg)	15 (12.4%)	12 (31.5%)
Cefepime (30 µg)	0 (0.0%)	0 (0.0%)
Cefoxitin (30 µg)	14 (11.6%)	1 (2.6%)
Aztreonam (30 µg)	0 (0.0%)	0 (0.0%)
Meropenem (10 µg)	0 (0.0%)	0 (0.0%)
Ertapenem (10 µg)	0 (0.0%)	0 (0.0%)
Gentamicin (10 µg)	0 (0.0%)	0 (0.0%)
Amikacin (30 µg)	0(0.0%)	0 (0.0%)
Ciprofloxacin (5 µg)	3 (2.5%)	3 (7.9%)
Pefloxacin (5 µg)	3 (2.5%)	3 (7.9%)
Ofloxacin (5 µg)	0 (0.0%)	0 (0.0%)
Levofloxacine (5 µg)	0 (0.0%)	0 (0.0%)
Tetracycline (30 µg)	1 (0.8%)	0 (0.0%)
Doxycycline (30 µg)	1 (0.8%)	0 (0.0%)
Trimethoprim-Sulfamethoxazole (1.25/23.75 µg)	1 (0.8%)	0 (0.0%)
Chloramphenicol (30 µg)	0 (0.0%)	0 (0.0%)
**ATB class resistance profile**		
Penicillin	2 (1.7%)	0 (0.0%)
Cephalosporin	29 (24.0%)	14 (36.8%)
Cyclines	1 (0.8%)	0 (0.0%)
Quinolones	3 (2.5%)	3 (7.9%)
Sulfonamide	1 (0.8%)	0 (0.0%)
**Multidrug resistance**	2 (1.7%)	0 (0.0%)

N_1_: Number of humans positive for *Salmonella* in this study. N_2_: Number of poultry positive for *Salmonella* in this study. ATB: Antibiotics.

**Table 4:** Prevalence of resistance and virulence genes in *Salmonella enterica* in human and poultry.

		**Human N_1_ = 121**	**Poultry N_2_ = 38**
**Resistance Genes**	**Functions**		
*aac(6’)-Iaa*	cryptic aminoglycoside Resistance.	121 (100.0%)	38 (100.0%)
*fosA7*	Fosfomycin Resistance	14 (11.6%)	5 (13.2%)
*qnrB19*	Quinolone resistance	1 (0.8%)	0 (0.0%)
**Virulence genes**	Pathogenicity island	**N_1_ = 121**	**N_2_ = 38**
*SPI1to5*		118 (97.5%)	34 (89.5%)
*SPI8*		22 (18.2%)	7 (18.4%)
*SPI9*		121 (100.0%)	38 (100.0%)
*SPI10*		2 (1.7%)	0 (0.0%)
*SPI12*		6 (5.0%)	2 (5.3%)
*SPI13*		79 (65.3%)	14 (36.8%)
*SPI14*		77 (63.6%)	14 (36.8%)
*invA*	Gene encoding fimbriae (or pili) and type III secretion systems (T3SS)	121 (100.0%)	38 (100.0%)
*invB*		121 (100.0%)	38 (100.0%)
*invC*		121 (100.0%)	38 (100.0%)
*invE*		121 (100.0%)	38 (100.0%)
*invF*		121 (100.0%)	38 (100.0%)
*invG*		121 (100.0%)	38 (100.0%)
*invH*		121 (100.0%)	38 (100.0%)
*invI*		121 (100.0%)	38 (100.0%)
*invJ*		121 (100.0%)	38 (100.0%)
*fimC*		121 (100.0%)	37 (97.4%)
*fimD*		121 (100.0%)	38 (100.0%)
*fimF*		87 (71.9%)	32 (84.2%)
*fimH*		118 (97.5%)	38 (100.0%)
*fimI*		121 (100.0%)	38 (100.0%)

N_1_ = total strain from human, N_2_ = total strain from poultry.

**Table 5:** Risks factor associated with *Salmonella* carriage.

**Carriage of *Salmonella enterica* in Humans**		**Odds Ratio Calculation**			
**Participant Characteristics**		**OR (95%CI)**	***p*-Value**	**aOR (95%CI)**	***p*-Value**
**Villages**	Basziri	Ref		Ref	
	Goulouré	0.7 (0.3–1.9)	0.516	0.7 (0.3 -1.9)	0.546
	Gouroumbila	0.8 (0.2–2.9)	0.679	0.8 (0.2–3.2)	0.761
	Nanoro	1.5 (0.7–3.2)	0.278	1.6 (0.7–3.5)	0.243
	Nazoanga	0.7 (0.3–1.5)	0.317	0.7 (0.3–1.5)	0.362
	Poessi	0.9 (0.4–2.4)	0.907	0.9 (0.4–2.4)	0.909
	Sitaon	0.3 (0.1–1.1)	0.050	0.3 (0.1–1.1)	0.071
	Zimidin	0.3 (0.1–0.9)	0.036	0.3 (0.1–0.9)	0.036
**Age category**	[0–10]	1.6 (0.5–4.5)	0.402	1.7 (0.6–5.0)	0.316
	[11–20]	2.4 (0.8–7.0)	0.116	2.8 (0.9–8.4)	0.066
	[21–30]	2.5 (0.7–8.9)	0.140	3.1 (0.8–10.9)	0.081
	[31–40]	Ref		Ref	
	[41–50]	2.2 (0.7–7.1)	0.176	2.0 (0.6–6.4)	0.253
	[51–60]	3.3 (1.1–10.2)	0.040	2.8 (1.0–8.9)	0.050
	[61–70]	1.6 (0.4–6.0)	0.466	1.3 (0.3 -4.9)	0.701
	[71–100]	0.4 (0.0–3.6)	0.406	0.3 (0.0–2.6)	0.262
**Sex**	Female	Ref		Ref	
	Male	1.2 (0.8–1.7)	0.410	1.2 (0.8–1.7)	0.394
**Seasons**	Dry season	Ref		Ref	
	Rainy season	1.8 (1.2–2.9)	0.008	1.6 (1.0–2.7)	0.047
**Carriage of *Salmonella enterica* in animals**					
**Characteristic determining factors**		**OR (95%CI)**	***p*-value**	**aOR (95%CI)**	***p*-value**
**Villages**	Basziri				
	Goulouré				
	Gouroumbila				
	Nanoro	1.3 (0.4–4.5)	0695	0.8 (0.2–4.1)	0.849
	Nazoanga	0.6 (0.1–2.7)	0.495	0.9 (0.1–5.9)	0.888
	Poessi	1.8 (0.4–7.5)	0.452	2.0 (0.4–11.1)	0.435
	Sitaon	0.2 (0.0–2.3)	0.209	0.3 (0.0 -4.3)	0.402
	Zimidin				
**Season**	March	0.5 (0.1–3.6)	0.480	0.5 (0.1–4.1)	0.485
	April	Ref		Ref	
	May	5.0 (1.0–25.4)	0.050	5.3 (0.9–30.4)	0.062
	June	1.1 (0.2–4.6)	0.970	0.9 (0.2–4.8)	0.888
	July	4.1 (0.9–24.6)	0.051	4.4 (0.7–28.5)	0.117
					
**Poultry ownership**	Number of heads	0.9 (0.9–1.0)	0.130	0.9 (0.9–1.0)	0.358
**Poultry treat**	No				
	Yes				
**Use of ATB to treat**		1.8 (0.2–13.5)	0.548		
	NA				
	No	Ref		Ref	

ATB: Antibiotics, aOR: adjusted odds ratio. CI: Confidence interval; wide CIs indicate limited precision due to small sample size or few events.

## Data Availability

All data (BioSamples, reads, and genome assemblies) for this study are available via NCBI under BioProject PRJNA1400071.

## Supplementary Materials

The following supporting information can be downloaded at: https://www.mdpi.com/article/10.3390/microorganisms14020294/s1, Table S1: Summary of bioinformatics analyses, tools, and parameters; Table S2: Point mutations associated with quinolone resistance detected by PointFinder.

## Appendix A. Blse-Salmonella Survey Questionnaire

1. Do you have antibiotics at home? (Check all medications available to the participant at home at the time of the survey to ensure that they are antibiotics or not)
  - Yes
  - No
2. If yes, specify the name.............
3. Have you used antibiotics in the last three (03) months? (Present to the participants the medications you have so that they can show the medications they have used)
  - Yes
  - No
  - Don’t know
4. If so, were these leftover antibiotics used to treat ongoing episodes of illness, or antibiotics purchased to treat each episode in their own right?
  - Use of remaining antibiotics
  - Antibiotics purchased to treat each episode in its own right
  - Don’t know
5. Have you visited any formal health care providers (ASBC, CSPS, CM, CMA, CHR, CHNU, Private Clinic) here or elsewhere in the last three (03) months?
  - Yes
  - No
  - Don’t know
6. Have you visited informal health care providers (medicine vendors at the market, medicine vendors in shops and kiosks, street medicine vendors) here or elsewhere in the last three (03) months?
  - Yes
  - No
  - Don’t know
7. Have you purchased antibiotics directly without prior consultation with a formal health worker (nurse and/or doctor) to treat one or more episodes of illness in the last three (03) months?
  - Yes
  - No
  - Don’t know
8. Have you been hospitalized in a formal health center (CSPS, CM, CMA, CHR, CHNU, Private Clinic) here or elsewhere in the last three (03) months?
  - Yes
  - No
  - Don’t know
9. What is your source of drinking water
  - Taps/Fountain taps
  - Drilling
  - Well built (protected from external contamination)
  - Undeveloped wells
  - Surface water (backwaters, dams, rivers, etc.)
  - Other (to be specified): .....................................
10. Do you have livestock?
  - Yes
  - No
11. If so, are they kept inside or outside the dealership?
  - Inside the concession
  - Outside the concession
12. How often have you consumed raw food (lettuce, cabbage, carrot, fresh unpasteurized cow’s milk) in the last three (03) months? |__|___
13. Do you wash hands with soap before every meal?
  - Yes
  - No

## Appendix B. Collection of Additional Data Asymptomatic Ebl-Salmonella Carriage

**Questionnaire addressed to the head of household**	
**Village: .....................................**	**Collection Date** :...../...../......
	**Collection time**: ......**:**......
**ID Ménage:** |___|___|___|___|___| - |_**V**_|	

1. Have poultry in your household
  |___| Yes |___| No
2. If so, how much head do you have approximately: ____________________
3. If so, do you treat them when they are a cause of illness?
  |___| Yes |___| No
4. If so, what do you use to treat them?
  Antibiotic? |___| Yes |___| No
  Another product to treat poultry? |___| Yes |___| No
  If which: ____________________
5. Can we take feces from any of them if you have them?
  |___| Yes |___| No
6. feces sampling done?
  |___| Yes |___| No
7. If the sample is not taken, state the reason: ____________________

Initial and Signature Agent:...................
