# Supplementary material for: Asymptomatic Carriage and Antimicrobial Resistance of Salmonella in Humans and Poultry in Rural Burkina Faso: Phenotypic and Genotypic Profiles and Associated Risk Factors
Source: Microorganisms. 2026 Jan 27;14(2):294. doi: 10.3390/microorganisms14020294 (PMC12943543; doi:10.3390/microorganisms14020294)
Supplement: Supplementary file 1 [file microorganisms-14-00294-s001.zip › microorganisms-4057017-supplementary.pdf]

Supplementary Table S1: Summary of bioinformatics analyses, tools, and parameters.

Analysis step	Tool	Version	Key parameters / database	Purpose
Read processing	BBMap (BBDuk, Tadpole)	v38.26	Default settings	Adapter trimming, low-quality read filtering, and error correction
De novo genome assembly	SKESA	v2.4.0	Default settings	Genome assembly from Illumina reads
Assembly polishing	Pilon	v1.23	Default settings	Error correction of assembled genomes
Assembly quality assessment	QUAST	v5.0.2	Default settings	Evaluation of assembly metrics
Taxonomic assignment	Mash	v2.2.1	RefSeq v93; identity >0.8; exclusion of phage/plasmid hits	Taxonomic classification of assemblies
Multilocus sequence typing (MLST)	MLST	v2.23.0	EnteroBase v2023-11-20; scheme senterica_achtman_2	Sequence type determination
Serovar prediction	SISTR	v1.1.3	--qc option; default settings	Salmonella serovar identification
Antimicrobial resistance genes	ResFinder	v4.3.1	DB v2023-05-25; coverage ≥60%, identity ≥90% (-l 0.6, -t 0.9)	Detection of acquired AMR genes
Pathogenicity islands	SPIFinder	v2.0	DB v2020-12-04; coverage ≥60%, identity ≥90% (-l 0.6, -t 0.9)	Identification of Salmonella pathogenicity islands
Virulence genes	ABRicate	v1.0.1	VFDB v2023-11-04; default settings	Identification of virulence-associated genes

Supplementary Table S2: Point mutations associated with quinolone resistance detected by PointFinder

Mutation	Nucleotide change	Amino acid change	Resistance
gyrA p.D87N	GAC -> AAC	D -> N	Confer resistance to quinolones
gyrA p.S83F	TCC -> TTC	S -> F	confer resistance to quinolones
gyrA p.S83F	TCC -> TTC	S -> F	confer resistance to quinolones
gyrA p.D87Y	GAC -> TAC	D -> Y	confer resistance to quinolones
gyrA p.S83F	TCC -> TTC	S -> F	confer resistance to quinolones
gyrA p.S83F	TCC -> TTC	S -> F	confer resistance to quinolones
gyrA p.D87N	GAC -> AAC	D -> N	confer resistance to quinolones
gyrA p.S83F	TCC -> TTC	S -> F	confer resistance to quinolones
gyrA p.S83F	TCC -> TTC	S -> F	confer resistance to quinolones
gyrA p.D87Y	GAC -> TAC	D -> Y	confer resistance to quinolones
gyrA p.S83F	TCC -> TTC	S -> F	confer resistance to quinolones
gyrA p.S83F	TCC -> TTC	S -> F	confer resistance to quinolones
parC p.T57S	ACC -> AGC	T -> S	not always translated to phenotypic resistance
